# Supplementary material for: Are carbon emissions trading and green financial instruments synergistic? -Comprehensive quantitative research based on content analysis
Source: PLoS One. 2024 Mar 7;19(3):e0298601. doi: 10.1371/journal.pone.0298601 (PMC10919601; doi:10.1371/journal.pone.0298601)
Supplement: S1 Data — (ZIP) [file pone.0298601.s001.zip › Data/地级市Z_F工作报告.zip!/║╙─╧/╔╠╟≡╩╨/└┤╫╘╣1⁄2╓┌║┼í╛┬φ┐╦╩2╛▌═°í┐_2013.docx]

各位代表：

现在，我代表市人民政府，向大会报告工作，请予审议，并请各位政协委员提出意见。

一、去年政府工作回顾

2012年，在省委、省政府和市委的正确领导下，面对复杂严峻的经济环境和繁重的改革发展稳定任务，全市上下深入贯彻科学发展观，认真落实省九次党代会和市四次党代会精神，加快实施《商丘市建设中原经济区承接产业转移示范市总体方案》，坚持“四个重在”实践要领，按照“持续求进、好中求快”的总基调，落实“三化”协调、粮为基础，“三新”推进、双轮驱动，四项重点、持之以恒，率先突破、打响品牌的总要求，不断加大经济运行调节力度，全市经济社会发展保持了比较好的趋势、态势和气势。全市生产总值实现1418．3亿元，增长11．2％，增速高于全省平均水平1．1个百分点，居黄淮四市第1位；规模以上工业增加值496．5亿元，增长17．1％，高于全省平均水平2．5个百分点，居全省第6位；固定资产投资1023亿元，增长23．9％，居全省第5位；公共财政预算收入70．2亿元，增长24．4％，居全省第4位；社会消费品零售总额546．1亿元，增长15．9％，居全省第5位；金融机构各项贷款余额705亿元，比年初增长19．8％；城镇居民人均可支配收入18312元，增长13．4％，增幅居全省第1位；农民人均纯收入6426元，增长14％，比全省平均水平高4个百分点，增幅居全省第3位，圆满完成了市四届人大一次会议确定的各项目标任务。

一年来，我们主要抓了以下八个方面的工作：

（一）落实调控政策，经济持续健康平稳发展。市政府高度重视经济运行工作，密切关注经济形势变化，积极跟踪政策走向，坚持每月召开一次经济运行工作分析会，研究解决经济运行中的问题，努力推动经济健康运行。针对经济运行中的瓶颈制约，我们认真落实省政府关于促进经济平稳较快增长的17条措施，加强对重点行业、重点企业、重点区域的运行调节，出台了29条关于支持60强和60高企业加快发展的政策措施，有效解决了影响企业发展的融资、用地、用工保障等难题。全年融资突破210亿元，新增贷款较年初增加116．6亿元，增量居全省第6位，增幅居全省第5位；申报各类建设用地3353公顷，供应国有建设用地565宗、1900．5公顷；培训企业在岗职工3．6万人，培养、引进高技能人才1．6万人。始终把调结构和稳增长摆在同等重要位置，坚持在发展中调整，在调整中提升，产业产品结构不断优化。二、三产业增加值占生产总值的比重达到77．2％，比上年提高1．4个百分点。全市规模以上工业企业主营业务收入2043．7亿元、利润总额115．6亿元。六大高成长性产业增加值完成211．9亿元，占全市规模以上工业增加值的比重达到42．7％，比上年提高4个百分点。产业集聚区发展势头强劲，全市产业集聚区完成固定资产投资747．6亿元，增长25．9％，其中工业项目完成投资568．7亿元，增长27．8％，占全市工业投资额的86．2％，基础设施投资完成83．7亿元，生产、生活配套设施逐步完善，承载功能进一步提升。产业集聚区内“三上”企业实现营业收入1400亿元，增长30．3％，制冷家电、超硬材料、纺织服装、煤化工、电动车等主导产业集聚集群效应逐步显现，产业集聚区已成为我市经济发展新的增长极。高度重视品牌质量建设，获得中国名牌产品3个、中国驰名商标3个，永城市产业集聚区被国家质检总局授予“全国知名品牌创建示范区”，全市质量总体水平明显提升。积极推动信息化建设，与中国移动河南分公司、中国电信河南分公司签署了“智慧商丘”建设战略合作协议，“两化融合”取得新进展。扎实开展环境保护工作，5条河流出境断面水质综合达标率93．6％，城市集中式饮用水源地水质达标率连续7年保持100％，城区空气质量优良率为92％，节能减排完成省定目标任务。

（二）优化区域布局，新型城镇体系日趋完善。以推进城乡统筹为主线，以商丘新区、商务中心区和新型农村社区建设为重点，加快构建现代城镇体系，城镇化水平进一步提高。全市城镇总人口达到353．6万人，城镇化率达到42．2％，比上年提高2个百分点。中心城区辐射带动作用不断增强。编制完成了商丘新区总体规划和商务中心区建设规划，中心城区控制性详细规划覆盖率达到80％以上。商丘新区310和105国道升级改造等重要交通项目顺利推进。商务中心区完成房屋征收128万平方米，收购储备土地1000余亩，融资规模超过20亿元，完成基础设施投资15亿元以上，宋城路、中州南路等主干道建设全面开工，日月湖引黄调蓄工程已启动。市政基础设施建设不断加强，投入中心城区基础设施建设资金113亿元，为历年来最多的一年。启动了长江西路、民主西路等道路新建和改造工程，开展了文化路、凯旋路、归德路、南京路等6条主干道环境综合整治。实施了包河景观带、2万吨污水处理和蔡河、忠民河、康林河等景观试验段工程，内河水系综合治理有效推进。完成了商丘和商丘东2个高速出入口整体改造等一批绿化项目建设，园林绿化水平明显提升。破解集体土地上房屋征收难题，中心城区旧城、城中村、棚户区改造面积262万平方米，启动城市社区开发项目37个。房地产业平稳健康发展，全年普通商品房竣工面积216万平方米，新开工面积273万平方米。

圆满完成了农村危房改造工作任务。创新市容环卫体制，建立了市、区分级清扫保洁模式，环境卫生作业质量和效果得到提升。坚持高起点、高标准推进县城规划建设，形成了各具特色的县域城区。加强中心镇与专业园区、新型农村社区建设的衔接，成功培育了一批产业鲜明的特色乡镇。新型农村社区建设快速推进，全面完成了全市新型农村社区规划，4618个行政村整合规划为716个新型农村社区，其中229个社区已经开工建设，新建住宅11．3万户，入住社区4．1万户，产业集聚区内新型农村社区基本完成年度建设任务。

（三）夯实“三农”基础，农业现代化进程不断加快。坚持把解决好“三农”问题作为全部工作的重中之重，进一步巩固农业的基础地位，努力实现农业增效、农民增收、农村稳定。组织实施了高标准粮田“百千万”工程，粮食生产再获丰收，总产达到125．9亿斤，比上年增长3．5％，实现了“九连增”，我市再次荣获全国粮食生产先进市称号。全年向国家贡献粮食34．8亿公斤，居全省第1位。畜牧业发展势头强劲，我市已成为国家重要优质畜产品生产加工基地。大力推进农业产业化集群和主食产业化培育工程，重点培育科迪、诚实人、贵友、中州食用菌等龙头企业，促进了面品、乳品、食用菌、辣椒等9个产业化集群发展。商丘农产品中心批发市场完成交易量713万吨，实现交易额187亿元。与杨凌农业高新技术示范区签署了现代农业发展框架协议，农业科技推广应用步伐加快。投入资金11亿元，加强农田水利基础设施建设，第18次荣获省“红旗渠精神杯”；实施农村安全饮水工程，解决了63．5万人的饮水安全问题；全市农机总动力达1161．6万千瓦，比上年增加21．3万千瓦；完成林业生态建设任务14．7万亩，居全省第1位，农村生产、生活、生态条件不断改善。

（四）强化工作措施，重点项目建设全力推进。深入开展“项目突破年”活动，持续完善工作机制，不断加强协调督导，认真谋划新区、铁路客货运枢纽、内陆口岸、高成长性产业和新兴产业等重大战略性基础性后备项目，有效地推进了重点项目建设。全市226个重点项目完成投资540亿元，增长29％。其中省管重点项目完成投资107亿元，占年度目标的157．8％。计划新开工项目135个，实际开工132个，竣工项目115个。碳纤维项目二期、冰熊制冷压缩机和压花铝板生产续建、香雪海家电科技二期、安踏鞋业一期、国药集团一期、应天海乐电子扩建、富士康实训工厂等项目已竣工或投产，济祁高速连霍至永登段建成通车，郑徐客运专线开工建设，商丘高铁站建设方案通过审批，沱浍河航运等项目建设取得较大进展。

（五）狠抓招商引资，区域发展优势持续增强。积极实施开放带动战略，按照“大项目～产业链～产业集群～产业基地”的思路，围绕主导产业，发挥自身优势，瞄准重点区域，紧盯重点企业，大力开展招商引资活动，积极承接国内外产业转移，招商引资成效显著。全市实际利用外商直接投资2．46亿美元，增长46．4％，增幅和完成任务比例居全省第3位；实际利用省外资金377．3亿元，增长28％，到位资金规模居全省第3位，增幅和完成任务比例居全省第2位。引进固定资产投资3亿元以上工业项目25个，总投资181亿元；引进1亿元至3亿元工业项目61个，合同资金183亿元。万宝电器、安踏产业园、博德铝业、中州电缆、大商新玛特、永安纺织、中国手套城、金锐金刚石、史丹利复合肥等一大批项目落地建设。成功举办了2012中国�商丘国际华商节，签约合同项目216个，总投资413亿元，进一步擦亮了“华商之都”品牌。

（六）推动科技创新，社会事业全面协调发展。把科技创新作为推动经济发展方式转变的基础性、战略性举措，制定奖励政策，实施强力推进。与中科院山西煤化所共同建设了“碳纤维新材料研发中心”，创建国家级企业技术中心1家、院士工作站1个，新认定国家级高新技术企业7家、省级工程技术研究中心7个，承担2项省级重大科技专项，民权、柘城产业集聚区被省政府批准为省级高新区。教育、卫生等社会事业全面发展，文化事业、文化产业进一步发展繁荣。新建、改扩建幼儿园170所，“两免一补”政策全部落实到位，永城职业学院和商丘幼师新校区开工建设，成功举办了“商丘杯”第五届黄河戏剧节，完成了第三次全国文物普查，大运河考古挖掘工作进展顺利，7个文化产业园区建设扎实推进。全年接待国内外游客1090万人次，实现旅游综合收入21．5亿元。深入开展群众性全民健身活动，成功举办了全市第四届运动会。加强人口和计划生育工作，人口自然增长率继续低于全省平均水平。

（七）保障改善民生，和谐商丘建设深入推进。始终坚持把民生改善作为政府工作的重心，全力统筹更多的财力、公共资源投向民生，真心实意为老百姓办实事、做好事。全市公共财政用于民生方面的支出达220．9亿元，占全市公共财政支出的77．1％。实施更加积极的就业政策，城镇新增就业人员7．8万人，城镇下岗失业人员再就业2．9万人，农村劳动力转移就业9．3万人。初步建立了覆盖城乡的社会保障体系，完成各类养老保险金征缴20．4亿元，发放28．2亿元；城镇基本医疗保险参保人数142．4万人；新农合参保人数712．8万人；城市低保月保障标准提高到260元，农村低保年标准提高到1500元；农村五保集中供养标准提高到每人每年2480元，分散供养标准提高到每人每年1500元。加大扶贫开发力度，11．2万农村贫困人口实现脱贫。争取保障性安居工程补助资金18．9亿元，新开工各类保障性住房3．6万余套，开工率、建设规模、争取资金均位居全省前列；全年归集住房公积金9．3亿元，发放住房公积金贷款2．2亿元。

（八）创新社会管理，民主法制和精神文明建设不断加强。坚持社会主义民主政治，自觉接受人大及其常委会监督，支持政协参政议政，办理省、市人大代表建议和政协提案915件，办结回复率100％，经征求代表委员意见，满意率和基本满意率在98％以上。坚持依法行政，全面落实重大行政决策合法性审查，市政府各类规范性文件、具体行政行为和重大合同均经法制机构审查，注重利用行政复议渠道化解行政争议。全面启动了“六五”普法工作，法制宣传教育和依法治市工作不断推进。深入开展群众性精神文明和文明城市创建活动，公民思想道德素质和城乡文明程度进一步提高。积极推进基层民主政治建设，第四届城市社区居委会换届选举工作圆满完成。坚持畅通群众信访渠道，积极解决群众反映的各种问题，及时化解社会矛盾，我市获得“全国信访系统先进集体”荣誉称号。完善公共安全应急管理体系，强化安全生产和食品药品监管，安全生产形势继续保持总体稳定，加强社会治安综合治理，人民群众安全感明显上升。深化源头治腐，加大腐败案件查处力度，政府系统党风廉政建设得到加强。加强审计监督，开展社会保障、安居工程、领导干部经济责任审计项目66个，涉及单位700余家，查出各类违规违纪资金20多亿元、管理不规范资金13亿元，向有关部门移交案件线索30余项。加强政府自身建设，深入开展“两转两提”和“三治三提”活动，政府职能和工作作风持续转变，行政效能和公务员素质不断提高。

坚持军民融合式发展，积极支持驻商解放军和武警部队建设，落实军人抚恤优待政策，做好军队转业干部和退役士兵就业安置、民兵预备役、人民防空以及拥军优属工作，军政军民团结进一步增强，连续第六次获得“全国双拥模范城”荣誉称号。统计、供销、工商、物价、海关、检验检疫、知识产权保护、盐业、烟草、邮政、妇女儿童保护、残疾人康复、外事侨务、对台、民族宗教、地方史志、档案、气象、防震减灾等各方面工作都取得了新的成绩。对口支援新疆巴里坤县工作进展顺利。

尤为可喜的是，去年以来，我们扎扎实实做了一些打基础、谋长远的工作，使商丘经济发展保持了速度加快、效益提升、后劲增强的良好势头。一是《商丘市建设中原经济区承接产业转移示范市总体方案》得到省政府正式批复。省政府赋予商丘许多含金量很高的支持政策，借此机遇我们及时与多家金融机构和省直部门签署了战略合作协议，为我市承接产业转移、加快对外开放、打造内陆开放高地赢得了多方支持。二是产业集聚区发展规划调整修编顺利完成。抢抓全省产业集聚区规划修编机遇，全市产业集聚区规划总面积由原来的156．6平方公里调整为286．4平方公里，新增129．8平方公里，进一步拓展了未来商丘发展空间。三是商丘新区和商务中心区规划建设全面启动。完成了商丘新区总体规划大纲编制，完善了新区管理体制，商务中心区规划建设强力推进，多个基础设施项目已经开工建设，新区和商务中心区建设迈出实质性步伐。四是一批重大基础项目先后立项批准。中石油商丘千万吨炼油项目已列入国家中原经济区发展规划，商丘机场项目已经通过国家发改委专家评估，进入最后的审批阶段，商合杭高铁已列入铁道部今年的规划，国电民权电厂二期工程初步可研已获省专家评审通过，神火集团商丘2�35万千瓦热电联产机组前期工作相关手续已上报国家能源局，我市持续发展的基础更加坚实、后劲持续蓄积。

各位代表，这些成绩的取得，令人鼓舞而又来之不易。这是省委、省政府和市委正确领导的结果，是市人大、市政协监督支持的结果，是全市广大干部群众团结奋斗的结果。在此，我代表市人民政府，向工作在各条战线上的工人、农民、知识分子、干部、人民解放军指战员、武警消防官兵、公安民警致以崇高的敬意！向所有关心、支持商丘发展的中央驻商单位、社会各界人士及海内外朋友表示诚挚的感谢！

各位代表！

总体上看，去年我市经济社会发展取得了明显成绩，但也存在一些不容忽视的困难和问题。一是经济社会发展还有诸多困难和挑战：经济基础仍然薄弱，经济总量偏小，人均水平较低；经济结构不优，煤电铝产业生产经营遇到前所未有的困难，煤炭行业价格、需求出现下降，效益下滑，电解铝行业仍然呈现亏损状态；经济环境复杂多变，不确定因素增多；生产要素制约依然存在，土地供需矛盾依然突出，中小企业融资难度加大，环境容量仍然有限，技术工人还满足不了企业发展的需求，财政保障能力有待进一步增强。二是政府工作还有许多亟待解决的问题：安全生产的一些环节、部位还存在隐患，安全生产特别是交通安全的监管手段尚需进一步创新和强化；城市建管水平、服务质量与广大市民的要求还有一定距离；一些干部的思想水平、业务素质、工作方法与新形势新任务的要求还不相适应，个别部门工作人员服务意识不强、工作效率不高、工作作风不实等现象仍然不同程度存在。对此，我们一定高度重视，努力在今后工作中逐步解决。

二、今年政府工作的总体要求和目标任务

今年是全面贯彻落实党的十八大精神和实施《中原经济区规划》的开局之年，也是我市建设中原经济区承接产业转移示范市大有作为的一年。做好今年的政府工作，对于落实好党的十八大、省九次党代会和市四次党代会精神，全力构筑中原经济区东部战略支撑，建设中原经济区东部门户，实现经济社会持续健康快速发展至关重要。

今年政府工作的总体要求是：深入学习和贯彻落实党的十八大和中央、省委经济工作会议精神，坚持以邓小平理论、“三个代表”重要思想、科学发展观为指导，以中原经济区建设为契机，以构筑中原经济区东部战略支撑为目标，以提高经济增长的质量和效益为中心，按照“持续求进、好中求快”的总基调，在加快信息化进程中持续探索“两不三新”“三化协调”科学发展路子，紧紧围绕主题主线，持续深化新型城镇化、产业集聚区、招商引资、项目建设四项重点工作，着力扩大投资、着力调整结构、着力改革开放、着力改善民生，努力建设中原经济区承接产业转移示范市，促进全市经济社会持续健康快速发展，保持社会和谐稳定。

今年全市经济社会发展主要目标是：生产总值增长11％左右，争取高于全省平均水平1～2个百分点；规模以上工业增加值增长17％；公共财政预算收入和全社会固定资产投资增速高于全省平均水平2个百分点以上；社会消费品零售总额增长15．5％；外贸出口增长15％；实际利用外商直接投资增长10％，实际利用省外资金增长15％；人口自然增长率控制在6．5％。以内；城镇居民人均可支配收入和农民人均纯收入增速高于全省平均水平1～2个百分点；居民消费价格涨幅控制在3．5％以内；节能减排控制在省定目标以内。

提出我市经济发展目标高于全省平均水平，主要基于以下两个方面考虑。一是无论经济总量还是人均水平，目前我市都处于全省较低水平，只有加快追赶速度，才能逐步赶上全省平均水平。二是从基础情况来看，我市已经具备较快发展的能力。近年来通过承接产业转移，建成了一批特色鲜明、具有市场竞争力的企业，特别是去年开工建设了一批重点工业项目，仅去年下半年就开工了总投资232亿元的49个工业项目，形成了新的经济增长点，今年还有112个工业项目相继开工建设，将为我市经济持续健康快速发展提供重要支撑，我们有信心、有决心、有能力实现全年经济发展目标。

为完成以上目标任务，今年着重做好以下八个方面的工作：

（一）围绕承接产业转移，加快构建现代产业体系。发挥资源丰富、要素成本低、市场潜力大的优势，积极承接国内外产业转移，加快推动经济转型升级，不断增强自我发展能力。培育主导产业，扩大经济总量。以建设“一谷三城六基地五中心”为目标，把主导产业作为承接转移的主体，延链条，补短板，做大总量，力争在集群式引进、板块承接、产业延伸上取得新突破。通过财税杠杆撬动、科研支撑等措施，扶植一批发展速度较快、经济效益较好、产品竞争力较强、发展潜力较大的重点骨干企业。引导、促进企业通过兼并重组、高位嫁接、强强联合、改制上市等多种方式做大做强。提升产业层次，优化现有存量。加快发展食品、纺织服装、装备制造、制冷等产业，加快推进商丘新型食品工业示范园建设，力争基础设施投资完成15亿元，入驻企业10家以上。改造提升煤炭、有色、化工、酿酒等传统产业，重点推进铝产品的精深加工，拉长产业链条。大力培育高成长性产业，抓好以碳纤维、超硬材料、特种玻璃为主的新材料产业，以生物制药、医疗器械等为主的新医药产业，以锂离子电池、环保设备为主的新能源产业，以“智慧商丘”和“三网融合”为重点的信息产业。壮大现代服务业，拓展支撑功能。积极发展楼宇特色经济，加快建设现代化的商务楼宇、商务景观、会展等设施，发展金融、中介、研发等产业。充分发挥豫东综合物流集聚区和商丘农产品中心批发市场、中意建材、光彩大市场等专业市场的平台作用，围绕纺织服装、电动车、食品、建材等支柱特色产业，规划建设仓储、配送、运输等社会化、现代化物流基地和专业市场，加快完善市场体系。加快发展文化旅游产业，着力提升特色文化产业品牌，推进商丘古城保护开发利用，培育黄河故道生态线林业、水上、乡村观光旅游。积极培育消费热点，发展连锁经营、电子商务等现代流通业和网上购物、网络教育等新型消费方式，发展养老服务、家政和社区服务等生活服务业。加快产业平台建设，做实发展基础。继续按照“四集一转”的要求，提升产业集聚区综合带动效应，力争全市产业集聚区完成固定资产投资800亿元，建成亿元以上工业项目100个以上，打造百亿元以上产业聚群5个。培育壮大主导产业集群，做大做强制冷、纺织服装、食品加工、新材料、电动车等产业，提升规模效应和集群集聚发展水平。加快道路、管网、通信等基础配套服务设施建设，完善检测、物流、金融、信息平台等公共服务功能，增强产业集聚区对项目和人才的吸引力。加大供电配网建设改造力度，重点做好产业集聚区电力保障能力建设，确保企业能够满负荷生产。

（二）围绕城乡统筹发展，持续推进新型城镇化进程。按照“一核两翼组团发展、四位一体统筹推进”的城镇化总格局，走集约、智能、绿色、低碳的新型城镇化道路。做强中心城区。注重规划引领，加快总体规划修编和中心城市组团式发展规划编制，启动新型城镇化发展规划编制。加大控规编制力度，力争中心城区控规覆盖率达到100％。注重功能完善，加快郑徐客专商丘站“三站合一”及交通换乘系统建设，优化市区路网结构，实施一批公园、道路绿化项目，积极推进污水、垃圾处理和内河水系治理等项目建设，着力解决交通拥堵、环境污染、城市内涝等突出问题。注重板块拓展，抓好商丘新区和商务中心区建设，积极推进新区南北主干道及郑庄、平台互通立交的建设。商务中心区按照“一年成名、两年成型、五年成城、八年成业”的要求，强力推进基础设施建设，重点抓好日月湖引黄调蓄工程、豫东大剧院和宋城路、中州路、睢阳路等市政道路的规划、设计和建设。加快商虞一体化进程，以道路互联互通为基础，实现规划、功能一体化。注重城区改造，进一步简化程序，提高效率，抓好旧城、城中村和棚户区改造与城市重点发展方向的结合，避免工作中的随意性和散发性。注重城市管理，推进相对集中行政处罚权工作，加快数字化城管平台建设，加大“双违”治理力度，集中整治出店经营、占道经营等脏、乱、差现象，努力做到城市管理的规范化、制度化、常态化。全面开展“六城”联创工作，通过广泛宣传发动，加强市民文明卫生素质教育、城市公益基础设施建设和道路街区环境综合整治，进一步巩固中国优秀旅游城市、国家园林城市、全国双拥模范城市创建成果，进一步深化国家卫生城市创建工作，启动国家森林城市、省级文明城市创建活动，全面提升城市综合形象。壮大县域城区发展实力。高起点、高标准地推进县城规划建设，统筹产业集聚区和县城发展，真正做到产城互动、产城融合，形成以城带产、以城促产、产城一体的格局。强化服务指导，在规划编制、拆迁安置、手续办理等方面给予支持，加快特色商业街区规划建设。加快小城镇建设。支持有一定特色产业、人口规模和基础条件的乡镇加快发展，培育一批工业型、商贸型、旅游型、特色农业型乡镇。扎实推进新型农村社区建设。重点推进城乡接合部、中心乡镇和有一定产业基础的特色乡村新型农村社区建设，新开工70个新型农村社区。整合各类涉农资金，加快100个试点社区的道路、电力、供排水、通信、公交车站等基础设施和教育、卫生、购物中心、文化娱乐等公共服务设施建设，完善社区功能，提高群众入住的积极性、主动性。探索建立有利于农村人口转移的户籍、土地、财税、社保等体制机制，加大城市基础设施、公共服务向农村延伸辐射力度，有序推进城乡一体化进程。

（三）围绕强化基础支撑，大力发展现代农业。紧紧围绕“粮食增产、农业增效、农民增收、农村繁荣”的发展目标，重点实施“三大建设工程”和“三大行动计划”。实施高标准粮田“百千万”建设工程。按照“稳定面积，主攻单产，改善品质，提高效益”的要求，新建高标准粮田126万亩，确保粮食综合生产能力稳定在120亿斤以上。继续实施“千名科技人员包千村”活动，推广农业先进技术和科研成果，确保良种覆盖率、测土配方施肥、病虫害专业化统防统治均达到100％，为粮食丰产丰收提供强有力的科技支撑。实施农业基础设施建设工程。以农田水利为重点，统筹推进水、电、路、林等田间生产设施建设，继续实施节水改造、小型农田水利重点县、农业综合开发、土地综合整治、新增千亿斤粮食产能规划工程，持续推进病险水库、水闸除险加固和中小河流治理工程建设，加快建设中部人工影响天气区域中心商丘基地项目，提高农业综合机械化水平，不断改善农业生产条件。实施现代农业产业化集群培育工程。结合农村土地流转，扎实推进农业产业化集群建设，培育主导产业，促进农业规模化经营。重点培育9个农业产业化集群，积极推进科迪、诚实人、贵友、中州食用菌等农业产业化集群试点发展，新培育5个面品、1个肉品和1个饮品农业产业化集群。实施农民收入倍增计划。调整种植业结构和农村经济结构，加快标准化养殖场区建设，大力发展现代畜牧业，增加农民家庭经营性收入；开展专业技能培训，提高农村劳动力素质和就业能力，增加农民工资性收入。同时，认真落实强农惠农政策，提高农民政策性收入；积极探索土地承包经营权、宅基地、农村房屋办证、抵押，变资产为资金，增加农民财产性收入。实施农民专业合作社发展计划。加大资金扶持力度，引导农业产业化龙头企业、经营性服务组织、基层供销社、返乡创业农民、各类专业大户和社会化服务组织等领办农民专业合作社，发展多种形式规模经营，构建集约化、专业化、组织化、社会化相结合的新型农业经营体系，新发展农民专业合作社600家。实施农村土地流转计划。按照依法自愿有偿的原则，鼓励和支持承包土地向种粮大户、家庭农场、农民合作社流转，实现“小块并大块”，新增农村土地流转面积40万亩，流转面积达到耕地面积的18％。

（四）围绕深化招商引资，着力打造内陆开放高地。加快中原经济区承接产业转移示范市建设，持续提升对外开放水平。抓好招商活动。认真组织参加第八届中博会、高交会、厦洽会、广交会等招商活动，瞄准长三角、闽浙粤、环渤海湾等重点区域，紧盯国际大企业，尤其是世界500强、行业500强企业，加强对接衔接，确保引进落地一批大项目、好项目。争取引进省外资金突破400亿元，利用境外资金突破3亿美元。引进3亿元以上项目30个，其中10亿元以上的特大项目争取达到10个。创新招商方式。围绕主导产业，突出专业化集群式招商，积极引进龙头企业和配套服务的中小企业，形成“雁阵效应”。充分发挥各类行业协会、商会的桥梁纽带作用，开展“以商招商”，鼓励企业通过战略投资、技术引进对传统产业进行改造提升。加强与沿海地区政府、产业园区、战略投资者的合作，探索以委托管理、联合开发、投资合作等方式共建产业园区。优化招商环境。建立招商引资和项目落地衔接机制，实行重点客商跟踪服务制度，真诚地对待客商，热情地接待客商，深入企业和项目工地现场办公，为客商提供一站式、全程式服务，打造低成本、高效率、无干扰的经济发展环境。完善开放平台。依托海关、出入境检验检疫等机构，加强与连云港、青岛等沿海港口的合作，加快公路、铁路、航空、海运、内河航运多式联运发展，完善快速大通关机制，打造内陆“无水港”。积极推动综合保税区建设，规划建设电子信息产业园，吸引国内外知名企业入驻。

（五）围绕重点项目建设，夯实跨越发展基础。继续实施项目带动战略，今年第一批计划安排重点建设项目253个，总投资1673亿元，当年计划投资527亿元，比上年增长28％。加快推进在建项目。确保总投资16亿元的金锐金刚石产业园标准化厂房竣工，分别投资12亿元的科信电缆一期、博德铝业一期、澳柯玛电器一期和总投资20亿元的君信化工项目建成投产，加快推进郑徐客专商丘段基础设施建设，总投资22亿元的港汇花园主体完工、总投资20亿元的开疆商务中心开工建设、总投资6亿元的凯泰国际汽配城完成主体建设。积极协调前期项目。对于神火集团2�35万千瓦热电联产机组、国电民权电厂二期工程、商丘机场、商合杭客运专线、邢口至商丘地方铁路等重大能源、工业、交通项目盯紧跟上，确保项目前期工作取得实质性进展，争取早日开工建设。开工建设郑民高速二期工程。启动石化园区规划，加快完善基础设施，积极引进配套产业，为中石油商丘千万吨炼油厂项目早日落地创造条件。主动作为争取项目。认真研究国家产业政策、产业发展方向和宏观经济走向，围绕主导产业，积极做好项目上报、审批等工作，争取有更多的项目进入国家、省计划盘子。把握机遇谋划项目。结合中原经济区规划和我市中原经济区承接产业转移示范市建设，认真谋划、论证一批工业、基础设施、民生保障等项目。在谋划和抓好项目发展上，力争重点项目投资占整个固定资产投资的70％以上，工业项目投资占整个重点项目投资的70％以上。强化服务支持项目。继续实施重点项目建设“五项机制”，按投资额对项目进行分级管理，开好工作例会，定期督导走访，及时协调解决征地、拆迁、资金等问题，严厉打击一切阻碍项目建设的不法行为。

（六）围绕增强发展活力，扎实推进改革创新。坚持以改革求突破、以创新促发展，破解要素瓶颈，优化资源配置，增强发展的动力和活力。推进重点领域改革。深化国有企业股份制、公司制改革和企业内部改革，支持神火集团参与省内外煤炭资源和铝产业的整合重组，继续深化与央企的战略合作。进一步完善国有资产监管体制，提高企业整体素质和资产质量，实现国有资产保值增值。鼓励、支持、引导非公有制经济发展，大力实施中小微企业成长工程。深化农村改革，推进集体林权和国有林场改革。积极推进财政、金融、价格、事业单位、文化体制、医药卫生体制等领域改革。创新土地资源保障机制。严格落实土地开发利用管理“三项机制”，积极稳妥探索人地挂钩政策，继续实行差别化土地调控政策，提高土地使用效率和土地供给能力。对列入省、市重点项目的招商引资项目，优先保障建设用地，对特别重大项目用地实行“点供”制。创新资金保障机制。继续开展“金融服务进两区”活动，搞好银企对接，引导金融机构加大对重点项目、重点行业、重点企业的支持。完善担保体系，提升融资担保能力，扩大社会融资总量，力争新增贷款不低于去年水平，资本市场融资90亿元以上，组建村镇银行2～3家，实现1～2家企业成功上市，引进1家股份银行。加强金融生态环境建设，培育诚实守信的社会信用文化，严厉打击恶意逃废债行为，规范民间融资行为，不断优化信用环境和金融环境。创新人力资源保障机制。改革人才培养模式，组织高校、高职院校和职业技能培训机构与用工单位对接，采取订单式培训和学工结合等形式，培养熟练工人，出台优惠就业政策，吸引人才本地就业。采取人才引进奖励措施，对引进的优秀人才给予专项资金奖励，提高其政治待遇，在全社会树立尊重知识、尊重人才的新风尚。创新科技体制机制。探索完善科技投入、绩效评价、成果转化等科技管理工作机制，完善科技创新奖励制度，激发各种科技创新要素的活力，逐步建立以企业为主体、市场为导向、产学研用紧密结合的技术创新体系，支撑、引领产业发展。力争新发展省级研发中心5家，争取国家、省科技计划项目10项，创建省级重点实验室、院士工作站和科技创新团队各1家。

（七）围绕生态环境建设，增强可持续发展能力。把节能减排和建设资源节约型、环境友好型社会放在更加突出的位置，保护生态环境，建设生态文明，推动可持续发展。强力推进节能减排。认真落实节能减排目标责任制，实行更加严格的环境准入和环境容量总量控制审批制度，开展排污权确认和交易工作，加快淘汰落后产能，严格控制能耗不合理增长，坚决实现节能减排目标。大力发展循环经济。加快推进循环经济试点建设，实施一批循环经济重大项目和示范工程，提升循环经济发展水平。依托优势资源，以产业集聚区和重点企业为载体，重点打造有色、煤炭、化工、农业和再生资源等领域循环产业链。切实加强环境保护。加快产业集聚区内污水处理设施建设，推进城区污水处理厂提标改造，加快垃圾处理、中水回用和污泥处理等设施建设。加强水源地保护，确保城区饮水安全。深入推进农村环境连片综合整治，统筹规划建设生活垃圾和污水处理设施，积极开展秸秆禁烧和农作物秸秆的综合利用。加快实施城区扬尘综合整治，规范建设工地施工，强化机动车环保检测，确保城乡空气、水质和声环境质量达到国家标准。继续抓好林业生态建设。重点抓好廊道造林、新型农村社区绿化、防护林建设等造林工程，加快退耕还林、天然林保护、植被恢复等生态建设工程。加强黄河故道生态林带建设，强化对城区公园、绿地、游园、道路生态走廊的建设和管护，继续实施内河水系绿化美化工程，努力建设“天蓝、地绿、水清、景美”的美丽商丘、生态商丘。

（八）围绕保障改善民生，不断提高公共服务水平。始终把保障和改善民生放在政府工作的首位，努力解决好人民群众最关心、最直接、最现实的利益问题，让人民群众得到更多实惠。加强就业再就业工作。实施全民技能振兴计划和“阳光工程”、“雨露计划”，努力提高劳动者素质，重点解决城镇零就业家庭、农村贫困家庭、“4050”人员和高校毕业生等群体的就业问题。发展各项社会事业。把教育摆在优先发展的战略地位，加大经费投入，促进教育资源均衡配置，统筹推进学前教育、基础教育、职业教育和高等教育。加大公共卫生投入，加强公共卫生服务体系建设，提升城乡医疗服务能力和技术水平。建立完善公共文化服务体系，开展丰富多彩的群众文化活动，加强文物保护和利用，加快推进大运河商丘段申遗工作。加强全民健身公共体育设施建设，广泛开展群众性体育活动。

继续稳定低生育水平，进一步完善利益导向政策，创建全国人口和计划生育综合改革示范市。完善社会保障体系。扩大城镇职工基本养老保险、基本医疗保险覆盖面，着力解决跨区域制度衔接问题，完善失业保险与城市低保制度的联动机制，提高城镇居民基本医疗保险、新型农村合作医疗、城乡低保和农村五保对象集中供养标准。启动城乡居民养老保险一卡发行工作，加快市县社会福利中心建设进度，推进慈善事业发展。推进平安商丘建设。加强和创新社会管理，突出防治结合，进一步完善严打长效工作机制，认真解决群众反映强烈的突出治安问题。全面落实安全生产责任制，增强公共卫生、食品药品安全、社会安全事件的预警和处置能力。健全综治信访维稳机制，畅通信访渠道，及时排查问题，有效化解矛盾，确保社会大局和谐稳定。

今年，我们将继续集中人力、财力、物力，办好事关人民群众切身利益的“十件实事”：一是推动劳动力就地转移就业。加强农民技能培训，加大公益性岗位购买力度，力争全年完成各类技能培训10万人，政府购买公益性岗位6000个，全市城镇新增就业6万人，下岗失业人员再就业2．1万人，新增农村劳动力转移就业9万人，其中就地转移就业5万人。二是关心救助弱势群体。扩大法律援助覆盖面，受理案件不低于3500件、接待咨询1．5万人次以上，实现应援尽援。提高低保标准，城乡低保人均月补差分别提高到200元、100元，农村五保集中供养标准提高到每人每年3200元，分散供养标准提高到2220元，把符合标准的重度残疾人纳入保障范围。三是改善农村生产生活条件。筹措资金2．5亿元，新改建农村公路200公里。筹措资金2．3亿元，解决50万农村人口安全饮水问题。四是加强保障性安居工程建设。开工建设各类保障性住房和城市棚户区改造28835套（户），其中廉租住房17659套，公共租赁住房8655套，城市棚户区改造2521户。争取国家和省补助资金1亿元，实施农村危房改造12730户。五是改善城区办学条件。加快城区学校建设，梁园区和睢阳区各建一所24班以上规模的标准化学校，加快商丘医专和商丘幼师新校区建设。六是加强城区基础设施建设。整修南京路（平原路～睢阳大道）非机动车道，其中南京路（平原路～包河段）铺设污水管网；完成6万平方米人防工程建设任务。七是推进城区生态环境治理。启动并加快运河综合治理，抓好运河上游蓝线以内房屋征收、土地整理和河道开挖工作，完成2万吨污水处理工程建设，改善城区生态环境。八是增强医疗卫生服务能力。开工建设市公共卫生服务中心项目，力争年底前完成主体工程建设。九是实施文化惠民工程。开展舞台艺术送农民和“百戏进百乡”活动，覆盖全市各乡镇；实施农村电影放映工程，确保全市每个行政村每月放映一场。免费开放博物馆、图书馆、群艺馆（文化馆）乡镇综合文化站等公益性文化设施。十是提高社会保障水平。新型农村合作医疗保险财政补贴标准由人均240元提高到280元，人均筹资水平达340元，参合率达97％。继续提高企业退休人员基本养老金，调整幅度不低于2012年人均养老金的10％。提高住房公积金缴存比例，实现全市财政供给人员住房公积金缴存比例不低于职工上年度月平均工资的5％，扩大非公有制企业住房公积金覆盖面。

深入开展国防教育和国防后备力量建设，支持驻商解放军和武警部队、预备役部队、民兵和人民防空建设，促进军民融合式发展；积极开展双拥共建活动，巩固和发展军政军民团结。着力推进民族团结进步创建活动，争创全国民族团结进步先进市。支持工会、共青团、妇联等人民团体开展工作，注重发挥工商联等社会团体作用。加强宣传组织，密切配合，认真做好全国第三次经济普查工作。继续做好外事侨务、妇女儿童保护、残疾人康复、地方史志、防震减灾等工作。

三、进一步提高推动科学发展的能力和水平

做好今年的政府工作，我们将认真落实中央关于改进工作作风、密切联系群众的八项规定，坚持用领导方式转变加快发展方式转变，深入推进“两转两提”和“三治三提”活动，进一步提高推动科学发展的能力和水平，努力建设人民满意的政府。

（一）科学决策，依法行政。建立健全重大决策的规则和程序，认真落实调查研究、专家论证、公众参与、合法性审查和集体决策制度，确保政府各项决策的科学性、民主性、合理性和实效性。认真执行人大及其常委会的决议、决定，自觉接受人大法律监督、政协民主监督，认真办理人大代表的议案、建议、批评、意见和政协委员的提案。严格按照法定权限和程序规定行使职权，加强和改进法制宣传、执法监督、行政复议、规范性文件制定和管理等政府法制工作。加强执法监察、效能监察、廉政监察，开展对职能部门履职情况监督检查，继续组织开展民主评议政风行风活动，广泛接受社会监督，进一步增强行政机关的服务意识，推进服务型行政执法建设，提高依法行政水平。

（二）以人为本，为民勤政。全市各级政府在推动经济和社会发展的进程中，要时刻牢记宗旨，始终把人民放在心中最高位置，坚持人民主体地位，把实现好、维护好、发展好最广大人民根本利益作为一切工作的出发点和落脚点，把群众赞成不赞成、高兴不高兴、满意不满意、拥护不拥护作为衡量各项工作的根本标准，把发展经济和改善民生结合起来，努力让人民群众成为改革发展的现实受益者。切实增强工作的责任感，脚踏实地，务实重干，把心思用在干事业上，把精力投到抓落实中，重实际、办实事、求实效，对于党委的决策，勇于担当，主动作为，抓关键，求突破，对于市人代会的决议，善思善谋，科学运作，不畏难、不推诿、不懈怠，最大限度地发挥职能作用，不断提高政府工作水平。

（三）转变作风，高效施政。树立正确的政绩观，坚持从发展实际出发，既立足当前又着眼长远，既积极进取又量力而行，不搞脱离实际的“政绩工程”和劳民伤财的“形象工程”，大兴求真务实之风，大兴调查研究之风，大兴埋头苦干之风，努力做出经得起实践、人民、历史检验的实绩。树立强烈的效率观，加快工作节奏，提高办事效率，加强督促检查，确保各项政策措施落到实处。继续改进文风会风，规范和精简公文，压缩会议，减少检查评比。深入开展机关效能建设，继续抓好政府审批制度改革，健全各项规章制度，全面推行政务公开，加快建立办事高效、运转协调、行为规范的行政管理体制。

（四）廉洁自律，从严治政。提倡艰苦奋斗，厉行勤俭节约，反对铺张浪费，珍惜民资民力，切实把有限的资金资源、财力物力用在发展经济和改善民生上。深入贯彻落实党风廉政建设责任制，深入开展警示教育，切实推进领导干部廉洁自律工作，规范权力运行，不断增强拒腐防变能力。坚持标本兼治、综合治理，建立健全监督和管理制度，推行领导干部经济责任和干部人事机构编制责任“双审计”制度，做到关口前移，从源头上遏制腐败现象。强化领导干部廉洁自律意识，严肃查处各种违反财经纪律的行为，加强对工程招投标、政府采购、国有资产转让等公共资源管理权的监督，树立良好的政府形象。加强公务员理论和业务学习，严格管理，完善录用聘用、交流轮岗、竞争上岗等制度，形成争先创优、优胜劣汰新机制，努力建设政治坚定、业务精通、清正廉洁、作风优良的公务员队伍。

各位代表！

商丘的发展已经站在了新的历史起点上，面对全市人民的新期盼，我们深感责任重大、使命光荣！让我们更加紧密地团结在以习近平同志为总书记的党中央周围，在省委、省政府和市委的正确领导下，紧紧依靠和团结全市人民，坚定信心，开拓创新，锐意进取，扎实工作，为实现经济社会又好又快发展，全面建成小康社会而努力奋斗！
